# A Case of Acoustic Shock with Post-trauma Trigeminal-Autonomic Activation

**DOI:** 10.3389/fneur.2017.00420

**Published:** 2017-08-16

**Authors:** Alain Londero, Nicolas Charpentier, Damien Ponsot, Philippe Fournier, Laurent Pezard, Arnaud J. Noreña

**Affiliations:** ^1^Service ORL et CCF, Hôpital Européen G. Pompidou, Paris, France; ^2^Faculté de médecine de Nancy, Université de Lorraine, Nancy, France; ^3^Lycée Germaine Tillion, Académie de Lyon, Sain-Bel, France; ^4^Laboratoire Neurosciences Intégratives et Adaptatives, UMR CNRS 7260, Fédération 3C, Aix-Marseille Université, Marseille, France

**Keywords:** tinnitus and hyperacousis, otalgia, pain, trigeminal nerve, acoustic shock, inflammation, referred pain

## Abstract

This study reports the case of an acoustic shock injury (ASI), which did not result in a significant hearing loss, but was followed by manifold chronic symptoms both within (tinnitus, otalgia, tingling in the ear, tension in the ear, and red tympanum) and outside the ears (blocked nose, pain in the neck/temporal region). We suggest that these symptoms may result from a loop involving injury to middle ear muscles, peripheral inflammatory processes, activation and sensitization of the trigeminal nerve, the autonomic nervous system, and central feedbacks. The pathophysiology of this ASI is reminiscent of that observed in post-traumatic trigeminal-autonomic cephalalgia. This framework opens new and promising perspectives on the understanding and medical management of ASI.

## Introduction

Acoustic shocks are brief exposure to loud sounds that do not cause substantial hearing loss but can trigger a cluster of debilitating symptoms, i.e., otalgia, ear fullness, ear tension, tinnitus, sound intolerance, dizziness and head, face or neck aches (1, 2). In most cases, these symptoms are temporary and disappear within a few hours or days following the acoustic incident. However, in certain cases, they can become chronic and seriously affect quality of life (1). The pathophysiological mechanisms underlying these symptoms remain unknown, even though some authors have hypothesized a dysfunction in the tensor tympani muscle (TTM) (1, 2). The patient described here was able to precisely report his symptoms, their temporal evolution, and take pictures of his eardrums over time during symptom severity fluctuations. The psychoacoustic characteristics of his tinnitus and the functional integrity of the middle ears were also investigated. This invaluable dataset provides critical insights into the pathophysiology of the acoustic shock injury (ASI) and beyond, i.e., tinnitus, hyperacusis, and otalgia.

## Methods

The patient (NC) was a 27-year-old Caucasian male working as a general medical practitioner at the time of the acoustic shock. Written informed consent was obtained from the participant for the publication of this case report. On November 10th 2013, in a leisure shooting stand, without wearing any hearing protection, he was exposed to a unique and unexpected gunfire at an approximate 7 m distance from his right side. As bothersome symptoms progressively emerged after this acoustic incident, he attended several ENT clinics. Audiograms at week 3 and 7 after ASI (thresholds <15 dBHL at all tested frequencies from 0.125 to 8 kHz) and all other exams (tympanometry, blood check, and cerebral MRI) were normal. Later, he was asked to assess the severity of his symptoms, using a 0–10 visual analog scale, for each ear several times a day (from May 1st to May 9th 2015). The symptoms were tingling, otalgia, ear tension, tinnitus loudness, neck pain/tension, temporal pain/tension, and pain/blocked nose. He also managed to photograph his eardrums using a video-otoscope (Firefly DE500 v1.1, 4 mm speculum). For analysis purpose, the images were transformed into numeric values estimating the “redness” of the eardrum. The number of red pixels was normalized according to the eardrum surface captured by the video-otoscope. In March 2017, NC visited our laboratory in Marseille to assess the psychoacoustic properties of his tinnitus and explored the middle ear function (Multifrequency Tympanometer Zodiac, Otometrics). Static admittance was obtained at four frequencies (226, 678, 800, and 1,000 Hz). Admittance variation while the patient voluntarily (but not forcefully) eye blinked was investigated over time both at the pressure at which the admittance is maximal and at ±50 daPa from this value. Symptom severity from each ear was analyzed using principal component analysis (PCA). The PCA was performed on all symptoms, excluding neck and temporal pain as these symptoms did not affect the left side.

## Results

**Figure 1:**
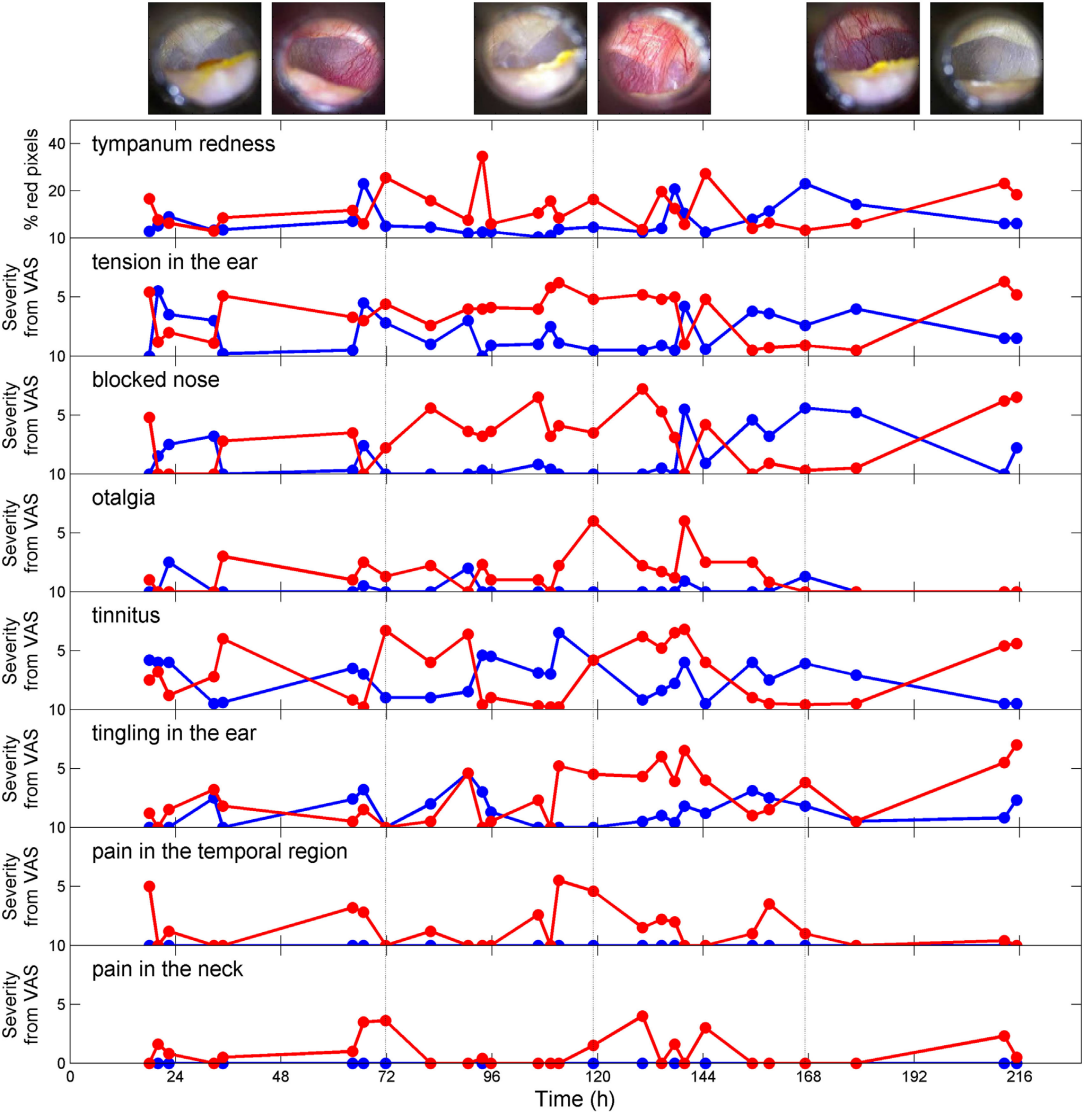
Severity of acoustic shock symptoms (assessed by VAS) as a function of time. The blue line represents the left ear and the red line represents the right ear. Examples of tympanum pictures are shown at the top (left images: left ear; right images: right ear, the vertical lines corresponds to the time at which the pictures were taken).

In regard to the clinical course, immediately after ASI, the patient felt a clicking in the right ear and thereafter reported a subjective perception of ear tension and fullness. At week 2 emerged a bilateral high-pitched fluctuating tinnitus. These auditory symptoms were associated with an erratic acute pain (sting or electrical shock) located deep in either ear. More than 3 weeks after the acoustic shock, the patient reported additional painful sensations starting around the concha and irradiating to the mid-face (constriction, blocked nose, and clear nasal discharge) and to the temporal region or the neck. Interestingly, the laterality and severity of the pain was correlated with the amount of tension perceived in the ipsilateral (right) ear. The psychoacoustic properties of the tinnitus, its amplitude modulation (tremolo), also varied according to the ear tension level. From low to mid tension, the tremolo varied from low frequency (“morse code”) to high frequency (“cricket-like sound”). For high tension, tinnitus was described as a high-pitched whistling. The pitch of this tinnitus was measured at 12 kHz and the tremolo, estimated from an amplitude modulated stimulus at the tinnitus frequency, was found at 32 Hz (the severity of ear tension and tinnitus loudness were 4 and 3, respectively). The loudness of this high-pitched tinnitus is reported in Figure 1. The patient also reported a low-pitched tinnitus associated with a sensation of fluttering in the ear. The low-pitched tinnitus was enhanced when something (earplug, stethoscope) was inserted in the ear canal. The high- and low-pitched tinnitus could be absent when the ear was completely relaxed. In contrast the tinnitus was not modulated by forceful head and neck contractions (3).

**Figure 2:**
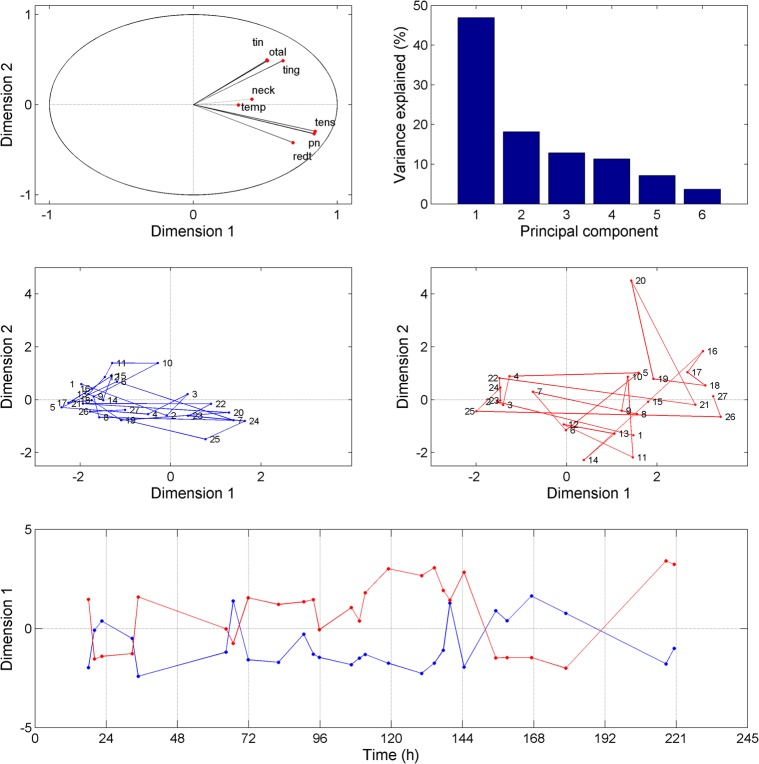
Results obtained from the principal component analysis (PCA). The upper left panel represents each symptom as a function of the first two principal components derived from the PCA. The upper right panel shows the percentage of variance explained by the different principal components. The middle panels (left: left ear, right: right ear) show the symptoms plotted at each time point as a function the first two principal components. The bottom panel shows the first principal component for each ear (blue line: left ear, red line: right ear) as a function of time.

The temporal evolution of symptoms severity (Figure 1) was investigated using PCA. The first principal component (48% of the total variance) represents the severity of the symptoms and does not discriminate between them. On the other hand, the second principal component (18% of the total variance) separates two symptom clusters, namely “tension,” “blocked nose,” and “tympanum redness” for symptom cluster 1 and “tingling,” “tinnitus,” and “otalgia” for symptom cluster 2 (Figure 2, upper panels). The feeling of pain in the temporal region and the neck (plotted on the first two PCA axes) represent a third group of symptoms (symptom cluster 3), different from cluster 1 and 2. The symptom cluster 3 is reported in the right side only, is moderate in severity, and is present only occasionally compared to the other symptoms. The PCA indicates that the correlation between symptom cluster 3 and both symptom cluster 1 and 2 is low. Figure 2 (middle panels) shows the multi-dimensional data (symptom severity) plotted on the new coordinate system defined by the first two principal components at each time point. The symptoms are usually more severe in the right ear than in the left ear, and the maximum severity is also greater in the right ear. Interestingly, the two symptom clusters are reported in the right ear, while only symptom cluster 1 is reported in the left ear. The temporal dynamics of symptom severity (first principal component) in the left and right ear (Figure 2, bottom panel) is also clearly anti-correlated across the two ears.

The sensation of tension in the ear was associated with tympanum hyperemia (Figure 1, also see Video S1 in Supplementary Material). This hyperemia was also present during jaw muscle contraction (hypothetically involving the mylohyoid muscle) and was associated with an increase of the high-pitch tinnitus (see Video S2 in Supplementary Material). This phenomenon was reported to be only present when the sensation of tension in the ear was high and coincidentally, the tympanum was always showing dilated vessels.

**Figure 3:**
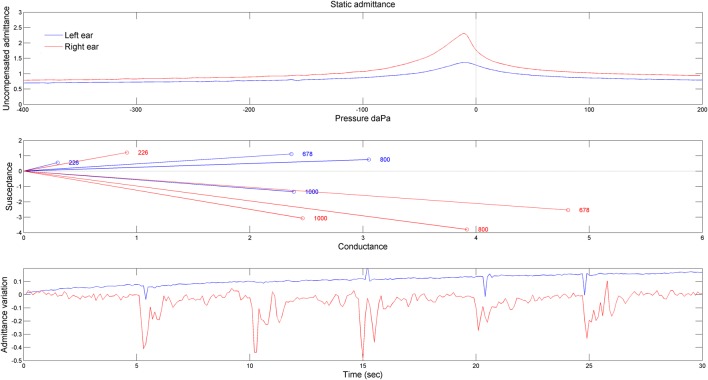
Results obtained by tympanometry measurements. The upper panel shows the static admittance for the two ears obtained for a 226 Hz probe. The middle panel shows the susceptance as a function of the conductance at four frequencies of probe tone stimulation (226, 678, 800, and 1,000 Hz). The bottom panel shows the admittance variation when the patient blinked voluntarily (but not forcefully) his eyes. The patient was asked to do so every 5 s (blue line: left ear, red line: right ear).

The tympanometry measurements demonstrated different middle ear function for the two ears (Figure 3). Indeed, for the right ear, the static admittance was larger (Figure 3, upper panel) and the resonant frequency (estimated from the frequency at which the susceptance is null) was abnormally low (<678 Hz) (Figure 3, middle panel). Moreover, the admittance was modulated by eyelid closure, especially in the right ear (Figure 3, bottom panel). Finally, the stapedial reflexes were present and clinically normal in both ears at 500 Hz and 4 kHz at 95–100 dB HL.

## Discussion

This report completes and adds to previous studies on acoustic shock (1). First, the symptoms reported after the acoustic shock, including their temporal dynamics and characteristics, have been described both qualitatively and quantitatively. This case reports all the symptoms that are commonly described after ASI, except vertigo and/or dizziness (1), and additional symptoms (nasal congestion, rhinorrhea, and tympanum hyperemia). Second, the functional integrity of the middle ears was also investigated using multifrequency tympanometry and direct examination of the eardrums. Our findings provide further insights into the mechanisms involved in ASI, and in particular they question and extend the framework previously developed by others (1, 2).

This study is the first to provide experimental support suggesting that middle ear muscles (MEM) can behave abnormally after ASI. Indeed, middle ear admittance is changed by simple eyelid closure, eardrum movements in the right ear can be large enough to be visible by the naked eye and the fluttering sensation (likely accompanying MEM contraction) is enhanced by cutaneous stimulation of the ear canal (4–7). This abnormal behavior of MEM may result from a dysfunction and/or injury of MEM caused by an exaggerated contraction in response to the acoustic incident. While stapedius muscle contraction is activated by loud sounds, TTM contraction can be triggered by unexpected sounds through the general startle reflex (8). The low resonant frequency and large admittance in the most symptomatic (right) ear may reflect an abnormal ossicle arrangement, possibly caused by the exaggerated contraction of the MEM. The sensation of tension in the ear reported immediately after the acoustic shock may result from an abnormal contraction of the MEM signaled by the muscle spindles (9) and tympanum mechanoreceptors due to eardrum deformation (10). The traumatic acoustic episode may lead to anxiety and sensory hypersensitivity, which may in turn reduce the startle reflex threshold and/or potentiate the startle reflex (11), possibly through a descending serotoninergic innervation (12). This central feedback may facilitate tonic and phasic TTM contraction.

The putative exaggerated contraction of the MEM, eventually followed by tonic contraction, may be associated with MEM injury and inflammatory processes. In particular, the TTM is innervated by fibers containing substance P and calcitonin gene-related peptide, which may play a key role in the noise-induced inflammation of the middle ear (13–15). Inflammation can then diffuse from the MEM to the tympanum and middle ear mucosa and can even become neurogenic as these tissues are rich in mastocytes (16–18). The diverse pain feelings in the ear (otalgia and tingling) likely result from middle ear inflammation and activation of the trigeminal nerve (TGN), which innervates the middle ear mucosa, the eardrum, and the TTM (15, 19). Intriguingly, tinnitus loudness is correlated to the severity of otalgia and tingling in the ear, suggesting that middle ear inflammation plays a role in tinnitus generation and/or severity. One can propose that antidromic activation of the TGN may change the vascular permeability of the blood vessels in the stria vascularis (20), which may eventually increase the endocochlear potential and produce tinnitus.

The MEM dysfunction/injury and the chronic inflammatory processes following ASI may be associated with neural hyperactivity in the trigeminal pathways. This neural hyperactivity may in turn lead to plastic changes (sensitization) at several levels of the trigeminal pathways (21). Pain in the neck and the temporal region may result from referred pain as a consequence of central sensitization, possibly at the trigemino-cervical complex (TCC) level where multimodal neurons are present (22). Sensitization may also contribute to shift the pathophysiology from acute to chronic (21).

Finally, the PCA suggests that the symptoms from cluster 1 originate from tightly coupled mechanisms. The trigeminal sensory inputs from regions of the head, including middle ear mucosa, eardrums, and the TTM (14, 15, 19), are collected by the TCC that has a reflex connection with the superior salivatory nucleus (SSN). Interestingly, the SSN provides a parasympathetic innervation to the head region *via* the sphenopalatine ganglion. This reflex loop between the TGN and the parasympathetic pathway of the head has been suggested to account for the autonomic symptoms associated with trigeminal-autonomic cephalalgia (TAC) (23). The activation of the trigeminal-autonomic reflex in this case may account for the nasal congestion, rhinorrhea, and the tympanic hyperemia. The autonomic nervous system, which innervates the middle ear (18, 24–26), may also contribute to enhance the MEM tonus and the feeling of tension and aural fullness (27).

In summary, this case suggests that a complex pathophysiology can be involved in ASI. Initially, the acoustic shock may trigger an exaggerated MEM response causing muscle dysfunction/injury and inflammatory processes, which may later diffuse to the tympanum and middle ear mucosa. The activation of the trigeminal pathways due to inflammation may account for the diverse pain feelings in the ear and tinnitus (symptom cluster 2) and also for referred pain in the neck and the temporal region (symptom cluster 3) after induction of central sensitization. Finally, nasal congestion and tympanum hyperemia, which are tightly coupled to the feeling of ear tension (symptom cluster 1), may result from the activation of a trigeminal-autonomic reflex. Interestingly, the pathophysiology of ASI is strongly reminiscent of that of post-traumatic TAC (28). In this context, the case reported here may define a new clinical entity that we suggest calling post-traumatic trigeminal-autonomic otalgia. The clinical picture presented here, i.e., with numerous, diverse, and severe symptoms, could be an extreme form of this clinical entity and may be relatively rare. However, the prevalence of this clinical entity may be more prevalent than expected at first sight, as less symptomatic forms may also exist. Many patients with tinnitus and/or hyperacusis report one or more additional symptoms such as ear fullness, otalgia, tympanic flutter, and/or pain in the neck (2). This framework opens new and promising perspectives on the understanding and medical management of ASI and beyond, i.e., tinnitus and hyperacusis (23).

## Ethics Statement

The case reported in the study gave written informed consent in accordance with the Declaration of Helsinki.

## Author Contributions

AL, NC, DP, PF, LP, and AN collected data. LP and AN analyzed data and made figures. AN wrote a first version of the manuscript. All authors contributed to the final version of the manuscript.

## Conflict of Interest Statement

The authors declare that the research was conducted in the absence of any commercial or financial relationships that could be construed as a potential conflict of interest.

## Supplementary Material

Video S1 The video shows the right eardrum when the feeling of tension in the ear is high and when it is low.

Video S2 The video shows the right eardrum, when the tension in the ear is high, before, during, and after the mylohyoid muscle contraction.
